# Depression as a Risk Factor for the Initial Presentation of Twelve Cardiac, Cerebrovascular, and Peripheral Arterial Diseases: Data Linkage Study of 1.9 Million Women and Men

**DOI:** 10.1371/journal.pone.0153838

**Published:** 2016-04-22

**Authors:** Marina Daskalopoulou, Julie George, Kate Walters, David P. Osborn, G. David Batty, Dimitris Stogiannis, Eleni Rapsomaniki, Mar Pujades-Rodriguez, Spiros Denaxas, Ruzan Udumyan, Mika Kivimaki, Harry Hemingway

**Affiliations:** 1 Department of Infection & Population Health, University College London, London, United Kingdom; 2 Farr Institute of Health Informatics Research, University College London, London, United Kingdom; 3 Department of Primary Care & Population Health, University College London, London, United Kingdom; 4 Division of Psychiatry, University College London, London, United Kingdom; 5 Department of Epidemiology & Public Health, University College London, London, United Kingdom; 6 Clinical Epidemiology and Biostatistics, School of Medical Sciences, Örebro University, Örebro, Sweden; Centro Cardiologico Monzino IRCCS, ITALY

## Abstract

**Background:**

Depression is associated with coronary heart disease and stroke, but associations with a range of pathologically diverse cardiovascular diseases are not well understood. We examine the risk of 12 cardiovascular diseases according to depression status (history or new onset).

**Methods:**

Cohort study of 1,937,360 adult men and women, free from cardiovascular disease at baseline, using linked UK electronic health records between 1997 and 2010. The exposures were new-onset depression (a new GP diagnosis of depression and/or prescription for antidepressants during a one-year baseline), and history of GP-diagnosed depression before baseline. The primary endpoint was initial presentation of 12 cardiovascular diseases after baseline. We used disease-specific Cox proportional hazards models with multiple imputation adjusting for cardiovascular risk factors (age, sex, socioeconomic status, smoking, blood pressure, diabetes, cholesterol).

**Results:**

Over a median [IQR] 6.9 [2.1–10.5] years of follow-up, 18.9% had a history of depression and 94,432 incident cardiovascular events occurred. After adjustment for cardiovascular risk factors, history of depression was associated with: stable angina (Hazard Ratio = 1.38, 95%CI 1.32–1.45), unstable angina (1.70, 1.60–1.82), myocardial infarction (1.21, 1.16–1.27), unheralded coronary death (1.23, 1.14–1.32), heart failure (1.18, 1.13–1.24), cardiac arrest (1.14, 1.03–1.26), transient ischemic attack (1.31, 1.25–1.38), ischemic stroke (1.26, 1.18–1.34), subarachnoid haemorrhage (1.17, 1.01–1.35), intracerebral haemorrhage (1.30, 1.17–1.45), peripheral arterial disease (1.24, 1.18–1.30), and abdominal aortic aneurysm (1.12,1.01–1.24). New onset depression developed in 2.9% of people, among whom 63,761 cardiovascular events occurred. New onset depression was similarly associated with each of the 12 diseases, with no evidence of stronger associations compared to history of depression. The strength of association between depression and these cardiovascular diseases did not differ between women and men.

**Conclusion:**

Depression was prospectively associated with cardiac, cerebrovascular, and peripheral diseases, with no evidence of disease specificity. Further research is needed in understanding the specific pathophysiology of heart and vascular disease triggered by depression in healthy populations.

## Introduction

Depression is a leading cause of disability worldwide and a major contributor to the global burden of disease.[1] The prevalence of depression in primary care settings has been shown to range between 5–10% in different countries.[2] Depression can have profound effects on social functioning, quality of life, and physical health through associations with cardiovascular disease, the leading cause of preventable death worldwide.[3] Meta-analyses of cohort studies suggest that depression is associated with 34–63% excess risk of all strokes combined[4–6] and 30–90% greater risk of aggregates of coronary heart disease.[6–9]

However, major clinical questions on the depression-cardiovascular disease hypothesis remain unanswered. Firstly, whether associations show any specificity across pathologically diverse cardiovascular diseases remains unclear. Cohort studies of depression have been too small to evaluate less common diseases (such as subarachnoid haemorrhage or abdominal aortic aneurysm)[10] or have lacked the clinical phenotyping[11] to report on common diseases such as heart failure and peripheral arterial disease. As a result, no studies have been able to directly compare the associations between depression and different cardiovascular diseases, which may inform understanding of disease mechanisms. Instead, many studies, including meta-analyses, have used aggregate endpoints [9,12,13] such as all types of stroke or all coronary heart diseases.[8,10] Secondly, the clinician needs to know whether patients who have a history of depression and patients diagnosed with new onset depression have similar or differential risks of subsequent cardiovascular diseases. This temporal resolution may point to different bio-behavioural mechanisms. Thirdly, the extent to which women experience higher or lower risk of any cardiovascular disease compared to men has not been reliably assessed. Although women consistently have between 1.5–2.5 times greater prevalence rates of depression compared to men,[14] previous investigations have been too small to examine gender modification in any associations with cardiovascular disease.[4] Lastly, there is a need to understand the extent to which associations can be explained by conventional cardiovascular risk factors. Cohort studies[11] as well as studies included in meta-analyses often have incomplete adjustment for covariates such as socio-economic status, smoking, blood pressure, cholesterol, and diabetes, [4,10] which may contribute to the high unexplained heterogeneity.

Answering these questions may contribute to the understanding of disease mechanisms and inform clinical practice. We used a large contemporary population-based cohort derived from linked electronic health records, including primary and secondary care, disease registry, and death records.[15] These large scale data provide a higher resolution of the depression exposure (distinguishing history and new onset) and of the outcome (distinguishing 12 chronic, acute, fatal and non-fatal, cardiovascular diseases.)

## Methods

### Data sources

Anonymised patients were selected from the CALIBER (CArdiovascular research using LInked Bespoke studies and Electronic Health Records) programme, described and validated elsewhere.[15,16] In brief, patients were linked across four clinical record data sources: the Clinical Practice Research Datalink (CPRD), the Myocardial Ischaemia National Audit Project (MINAP) registry, Hospital Episodes Statistics (HES) and disease-specific mortality. CPRD provides primary care data on clinical diagnoses, prescriptions, medical procedures, health behaviours, anthropometric measurements, and laboratory tests, using the Read clinical coding scheme[17], a hierarchical coding structure which maps onto ICD-10 codes and includes additional symptom and diagnostic codes. MINAP is a national registry of patients admitted to hospital with an acute coronary syndrome. HES provides information on diagnoses and medical procedures related to all elective and emergency hospital admissions across all National Health Service hospitals in England.

### Study Population

Patients registered from 225 general practices were studied using an open cohort design. Follow-up started on the date patients met the following eligibility criteria after 1 January 1997: age 30 years or older, at least one year of pre-study follow-up within a CPRD general practice, and free from clinically diagnosed cardiovascular diseases (any of the 12 cardiovascular outcomes studied, see ‘cardiovascular risk factors‘). We used each patient’s entire medical history available to confirm they were free from clinically diagnosed CVD. The look-back period ranged from 20 years to the minimum of 1 year, which has been shown to be a sufficient period to accrue accurate assessment of initial disease presentations.[18] Patients were censored on the date of first cardiovascular disease presentation, death, or the date of last data collection from the CPRD (20 March 2010), whichever occurred first.

**Fig 1:**
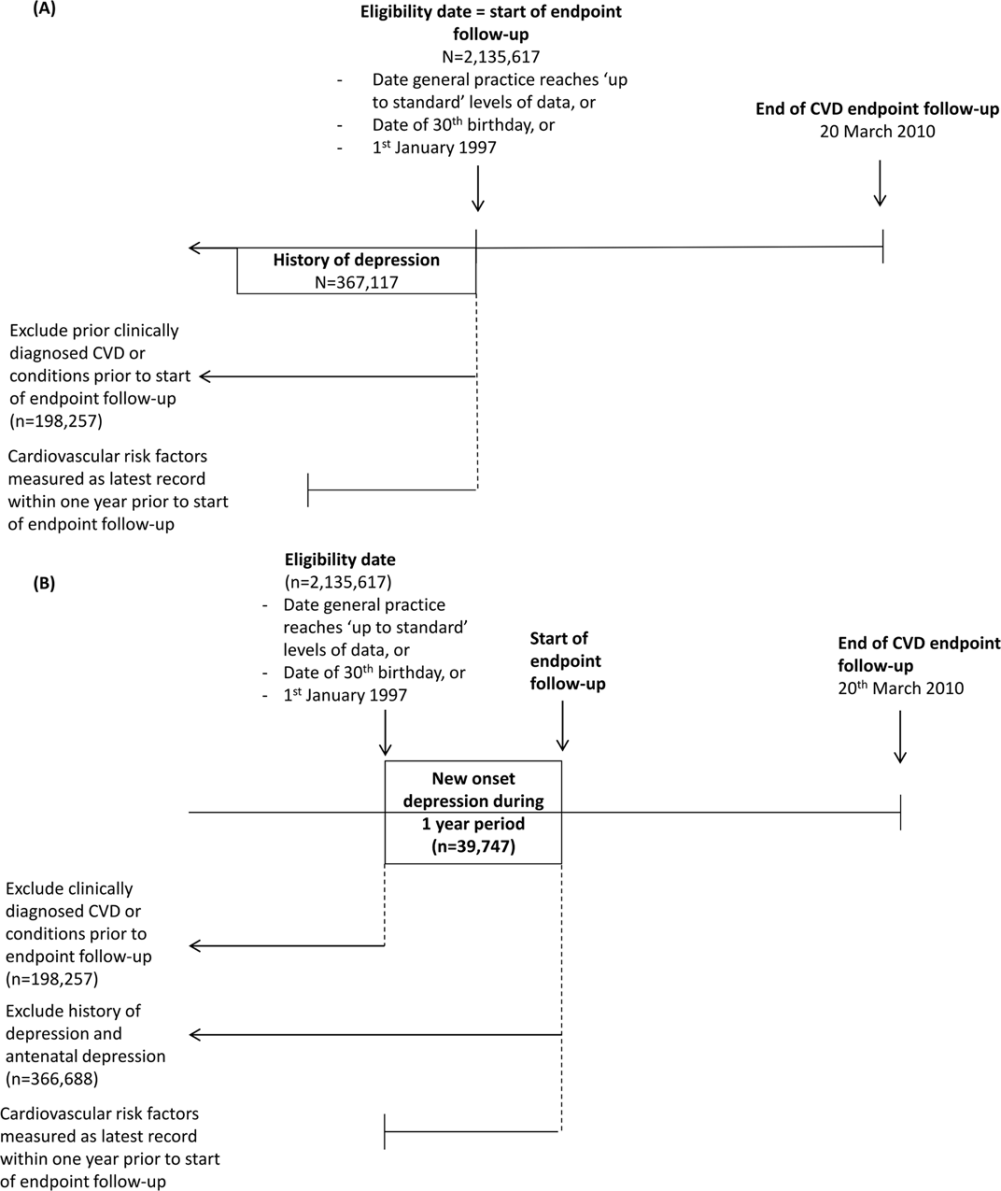
Cohort diagrams. (A) History of depression cohort (B) New onset depression cohort.

A total of 2,135,617 patients met the eligibility criteria during the study period; of those, 198,257 had prior clinically diagnosed cardiovascular diseases or conditions and were excluded.(See Table A in S1 File for detailed CVD definitions) Two cohorts were derived from this population of 1,937,360 remaining patients. (Fig 1)

### Cohort 1: History of depression

Among 1,937,360 patients, we defined history of depression as a CPRD record of a depression diagnosis and/or prescription of anti-depressant medications at any point prior to baseline. (Fig 1 Panel A) Those with no history of depression at any point prior to baseline were the reference group. Detailed information on specific Read codes used to define depression can be found in Text A in S1 File.

### Cohort 2: New onset depression

We defined a baseline period as the one year between the date when patients meet inclusion criteria and the beginning of cardiovascular endpoint follow-up. (Fig 1 Panel B) New onset depression was defined as a CPRD record of a depression diagnosis and/or prescription of anti-depressant medications (selective serotonin reuptake inhibitor, monoamine-oxidase inhibitor, tricyclic, or other antidepressants) during this baseline period. Patients with no evidence of depression during baseline were the reference group. Of the eligible 1,937,360, we excluded patients with records of depression diagnosis, history of depression, antenatal depression, or anti-depressant prescriptions (n = 366,688) up to and including baseline. A further 214,094 had clinically diagnosed cardiovascular events the year following CPRD registration or follow-up which did not extend to the cohort entry date, and were excluded. This derived a cohort of 1,356,578 patients.

### Cardiovascular risk factors

Socio-demographic and cardiovascular risk factors were obtained from CPRD, as recorded during consultations in primary care. We used the most recent measurement (blood pressure, lipids, body mass index) or prescription recorded up to one year prior to start of cardiovascular endpoint follow-up. Socioeconomic status was defined using quintiles of the index of multiple deprivation 2007, which provides a relative ranking of areas across England according to their level of deprivation.[19] Read codes were used to define health behaviours (smoking status, alcohol consumption.) We defined diabetes mellitus as prescription for oral hypoglycaemics or insulin, or a diagnosis of diabetes. Full covariate definitions are provided at http://www.caliberresearch.org/portal/.

### Cardiovascular disease endpoints

Our primary endpoints were the first recorded diagnosis of the 12 most common presentations of fatal or non-fatal cardiovascular disease (CVD) whether occurring in primary care, secondary care, or at death. We studied the following 12 CVDs: stable angina, unstable angina, non-fatal myocardial infarction (MI), unheralded coronary heart disease death (death with the primary cause certified as coronary heart disease, and no prior history of cardiovascular disease, including patients with myocardial infarction who died on the day of their infarct), heart failure, a composite of cardiac arrest, ventricular arrhythmia and sudden cardiac death (SCD), transient ischemic attack, ischemic stroke, subarachnoid haemorrhage, intracerebral haemorrhage, abdominal aortic aneurysm, peripheral arterial disease, in addition to composite CVD. We classified events as fatal where a death record existed for the same calendar date. We defined the end of follow-up at first occurrence of the first cardiovascular endpoint diagnosis, de-registration with the general practice, last data collection for the practice, or death from non-cardiovascular causes. (See Table A in S1 File for an overview of diagnostic codes and data sources used to define cardiovascular end points.)

### Statistical analysis

To compare incidence of each of the 12 CVDs in the cohort according to GP-diagnosed depression status at baseline, we derived cumulative incidence rates per 100,000 person-years, adjusted for the competing risk of presentations with other CVDs or death from other causes.

To derive hazard ratios (HR) and 95% confidence intervals for the association of depression and 12 CVDs, we used disease-specific Cox proportional hazards models.[20] Models were adjusted for age (linear and quadratic term), sex, social deprivation, smoking status, systolic blood pressure, total cholesterol, high-density lipoprotein, and diabetes, and stratified by primary care practice. To replace missing covariate data for variables not violating the missing at random assumption, we implemented multiple imputation using the *mice*[21] algorithm. (Full description Text B in S1 File). Imputation models were estimated separately for men and women and included baseline covariates, averages of continuous covariates within pre- and post-baseline periods of up to 5 years, as well as measurements of blood pressure, creatinine, and comorbid conditions. Five multiply imputed datasets were generated, and Cox models fitted to each dataset. Estimates were combined using Rubin’s rules. Assuming mutual independence between initial presentations, we assessed heterogeneity of the hazard ratios based on tau (τ^2^), a moment-based statistic of the residual between-group variance.[22] Analysis was performed using R version 15 for Unix and Stata 13.

### Sensitivity analyses

Due to missing data for ethnicity and alcohol consumption in the CPRD, we repeated analyses restricting the cohort to registrants with non-missing data (complete case.) Due to the known excess risk of CVDs associated with diabetes[23], we excluded participants with diabetes at baseline and repeated the analyses. In addition, all models were also repeated for new onset depression as well as history of depression with adjustment for body mass index (BMI), in addition to all other risk factors already adjusted for.

## Results

Of 1,356,578 patients in with records during the one year baseline period, 39,747 (2.9%) had a GP diagnosis of new onset depression. (Table 1) Over 63.0% were women, 92.8% identified as white, and 22.9% were in the lowest socioeconomic group. Individuals with new-onset depression had higher prevalence of smoking, diabetes, use of anti-hypertensive medications, and almost three-fold higher number of consultations in the past year compared to those without depression at baseline. Out of 1,937,360 patients with records prior to the one year baseline, 367,117 (19.0%) had a GP diagnosis of history of depression.

**Table 1:** Cardiovascular disease risk factors among people with and without new onset depression at baseline.

	No depression at baseline (n/N = 1,316,831/1,356,578)†		New onset depression at baseline (n/N = 39,747/1,356,578)		History of depression (n/N = 367,117/1,937,360)	
	n	(%)	n	(%)	n	(%)
**Women**	604,278	(45.9)	25,381	(63.9)	245,989	(67.0)
**Mean age, years (SD)**	47.3	(15.1)	47	(15.0)	48.3	(15.6)
Ethnicity*						
White	587,711	(90.4)	23,153	(92.8)	219,042	(59.7)
South Asian	19,151	(2.9)	593	(2.4)	4,077	(1.1)
Black	21,150	(3.3)	615	(2.5)	3,673	(1.0)
**Socioeconomic status**						
Least deprived	278,418	(21.1)	7,016	(17.7)	62,384	(17.0)
Most deprived	242,922	(18.4)	9,115	(22.9)	88,687	(24.2)
Smoking*						
Non smoker	45,142	(21.9)	1,853	(18.2)	11,706	(3.2)
Ex smoker	72,002	(34.9)	3,142	(30.8)	26,883	(7.3)
Current smoker	88,925	(43.2)	5,193	(51.0)	43,285	(11.8)
**Diabetes mellitus**	33,390	(2.5)	1,386	(3.5)	14,197	(3.9)
**Mean body mass index, kg/m**^**2**^ **(SD)**	26.3	(5.0)	26.5	(5.5)	27.0	(5.9)
Mean blood pressure*, mmHg (SD)						
Systolic	130.6	(19.1)	128.4	(18.8)	129.0	(19.0)
Diastolic	78.7	(10.2)	77.8	(10.2)	78.0	(10.4)
Mean lipid concentration*, mg/dL (SD)						
Total cholesterol	5.4	(1.1)	5.4	(1.2)	5.0	(1.2)
High density lipoprotein	1.4	(0.4)	1.4	(0.4)	1.0	(0.4)
**Medication use**						
Statins	30,217	(2.3)	1,275	(3.2)	15,682	(4.3)
Any blood pressure-lowering drug	178,605	(13.6)	7,671	(19.3)	-	-
**Mean number of consultations in last year (SD)**	3.8	(5.0)	10.0	(8.3)	8.0	(8.3)

Abbreviations: BMI, body mass index; DBP, diastolic blood pressure; HDL, high density lipoprotein; SD; standard deviation; SBP, systolic blood pressure

† This category refers to the reference group from cohort of new onset depression at baseline (total n = 1,356,578): n = 39,747 with depression versus n = 1,316,831 with no depression at baseline.

* Missing values in the whole sample (1,937,360) for ethnicity = 50.25%; smoking = 84.0%; systolic blood pressure = 60.2%; diastolic blood pressure = 60.2%; total cholesterol = 90.0% and high density lipoprotein = 95.0%.; Missing values in the study population (1,356,578) for ethnicity = 36.89%; smoking = 77.7%; systolic blood pressure = 50.0%; diastolic blood pressure = 50.0%; total cholesterol = 89.0% and high density lipoprotein = 92.7%.

Median (IQR) follow-up for CVDs was 6.9 (3.2–10.5) years. During 9,399,559 person-years at risk, a total of 27,311 incident coronary heart disease, 11,870 other cardiac, 14,580 cerebrovascular, 10,000 abdominal/lower limb disease events, and 38,765 non-cardiac related deaths were observed. As shown in Table 2, the highest incidence rates (>100 events/100,000 person-years at risk) for individuals with history of depression were found for myocardial infarction, stable angina, heart failure, transient ischemic attack, and peripheral arterial disease. Individuals with new onset depression had the highest incidence rates for the same CVDs as those with history of depression, with the exception of transient ischemic attack.

**Table 2:** Cumulative incidence rate (IR) of 12 cardiovascular diseases per 100,000 person-years at risk (PYR) among people with new onset depression at baseline and with history of depression prior to baseline.

	No depression at baseline (n/N = 1,316,831/1,356,578)†		History of depression (n/N = 367,117/1,937,360)		New onset depression at baseline (n/N = 39,747/1,356,578)	
	N events	IR/100,000 PYR	N events	IR/100,000 PYR	N events	IR/100,000 PYR
**Coronary**						
Stable angina	7,983	87	3,106	147	279	108
Unstable angina	3,439	38	1,536	73	137	53
Myocardial infarction	11,085	121	3,257	154	285	110
Unheralded coronary death	3,981	44	1,124	53	122	47
**Other myocardial**						
Heart failure	9,132	100	3,026	143	265	102
Cardiac arrest/sudden cardiac death	2,403	26	622	30	70	27
**Cerebrovascular**						
Transient ischaemic attack	7,538	82	2,643	125	247	95
Ischaemic stroke	4,168	46	1,312	62	119	46
Subarachnoid haemorrhage	819	9	315	15	34	13
Intracerebral haemorrhage	1,610	18	533	25	45	17
**Abdominal and lower limb**						
Peripheral arterial disease	7,423	81	2,661	126	261	101
Abdominal aortic aneurysm	2,261	25	550	26	55	21
**Total CVD**	74,821	819	25,247	1,198	2,322	898

† This category refers to the reference group from cohort 1 -new onset depression at baseline (total n = 1,356,578): n = 39,747 with depression versus n = 1,316,831 with no depression at baseline.

**Fig 2:**
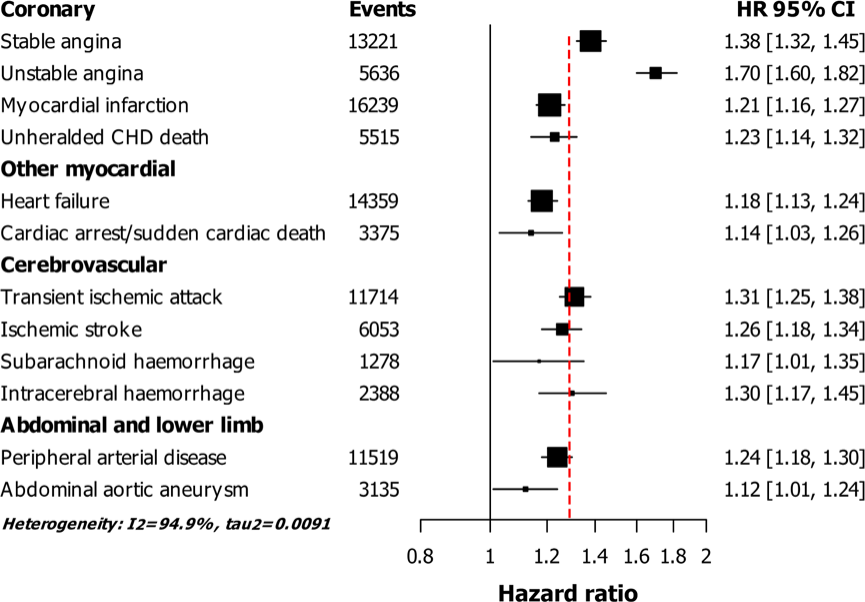
Hazard ratios (HR) and 95% confidence interval (95%CI) for the association of history of depression with 12 cardiovascular diseases, adjusted for age, gender, smoking, systolic blood pressure, diabetes, cholesterol, and socio-economic status (94,432 events in 1,937,360 men and women). Abbreviations: CHD, coronary heart disease; SCD, sudden cardiac death. Vertical dotted line indicates the HRs for total cardiovascular diseases: HR = 1.28 (95%CI 1.26–1.30).

After adjustment for cardiovascular risk factors, we found that history of depression was associated with higher risk of each of the 12 cardiovascular diseases; stable angina (HR = 1.38, 95%CI 1.32–1.45), unstable angina (1.70, 1.60–1.82), myocardial infarction (1.21, 1.16–1.27), unheralded coronary death (1.23, 1.14–1.32), heart failure (1.18, 1.13–1.24), transient ischemic attack (1.31, 1.25–1.38), ischemic stroke (1.26, 1.18–1.34), intracerebral haemorrhage (1.30, 1.17–1.45), and peripheral arterial disease (1.24, 1.18–1.30). (Fig 2) Weaker associations were found with cardiac arrest/sudden cardiac death, subarachnoid haemorrhage, and abdominal aortic aneurysm.

**Fig 3:**
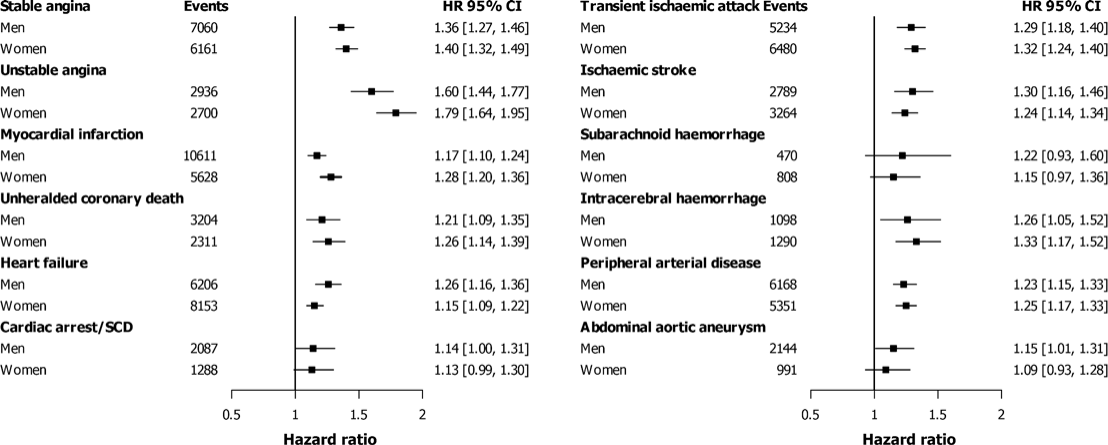
Hazard ratios (HR) and 95% confidence intervals (95%CI) by gender for the association of history of depression with 12 cardiovascular diseases, adjusted for age, smoking, systolic blood pressure, diabetes, cholesterol, and socio-economic status (94,432 events in 958,329 men and 979,031 women). **P-values for interaction between gender and history of depression: stable angina p = 0.618, unstable angina p = 0.174, myocardial infarction p = 0.210, unheralded coronary death p = 0.478, heart failure p = 0.101, cardiac arrest/sudden cardiac death p = 0.972, transient ischaemic attack p = 0.632, ischaemic stroke p = 0.113, subarachnoid haemorrhage p = 0.683, intracerebral haemorrhage p = 0.612, peripheral arterial disease p = 0.265, abdominal aortic aneurysm p = 0.303.** Abbreviation: SCD; sudden cardiac death.

We found no significant differences in covariate-adjusted risk of any cardiovascular endpoint between men and women with GP-diagnosed history of depression (Fig 3) or new onset depression (Figure A in S1 File).

**Fig 4:**
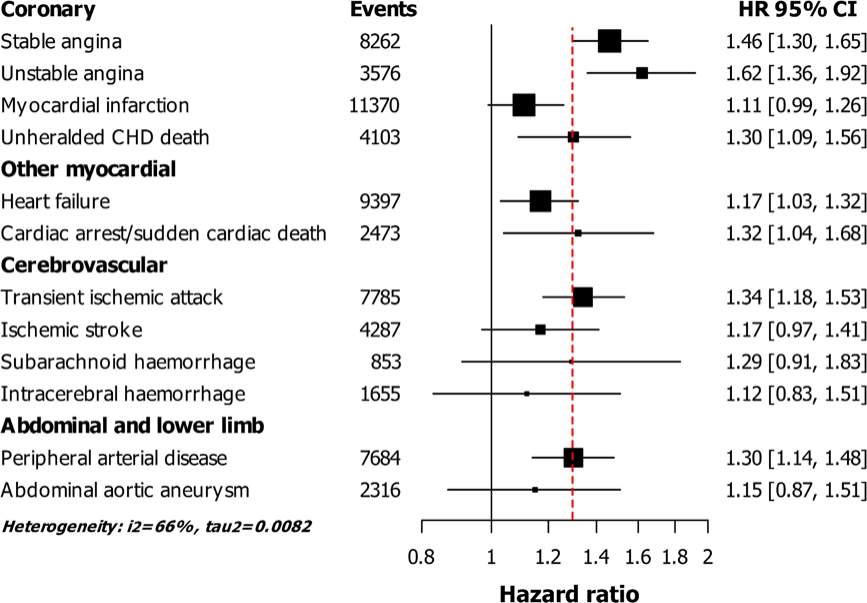
Hazard ratios (HR) and 95% confidence interval (95%CI) for the association of new onset depression with 12 cardiovascular diseases, adjusted for age, gender, smoking, systolic blood pressure, diabetes, cholesterol, and socio-economic status (63,761 events in 1,356,578 men and women). Abbreviations: CHD; coronary heart disease, SCD; sudden cardiac death. Vertical dotted line indicates the HRs for total cardiovascular diseases: HR = 1.30 (95%CI 1.24–1.35).

After adjustment for cardiovascular risk factors, we found that new onset depression was associated with stable angina (HR = 1.46, 95%CI 1.30–1.65), unstable angina (1.62, 1.36–1.92), unheralded coronary heart disease death (1.30, 1.09–1.56), heart failure (1.17, 1.03–1.32), cardiac arrest/sudden cardiac death (1.32, 1.04–1.68), transient ischemic attack (1.34, 1.18–1.53), and initial presentation with peripheral arterial disease (1.30, 1.14–1.48). (Fig 4) Weak associations were found between new onset depression and initial presentation of myocardial infarction, ischemic stroke, subarachnoid and intracerebral haemorrhage, and abdominal aortic aneurysm. The associations with all cardiovascular endpoints did not differ significantly between history of depression and new onset depression.

### Sensitivity analyses

Among participants with complete data for ethnicity and alcohol (n = 674,931 new onset depression, n = 1,018,538 history of depression), adjustment for these two factors, in addition to cardiovascular risk factors, did not alter the associations. (Figs B and C in S1 File) We repeated main analyses after exclusion of participants with diabetes at baseline (for history of depression n = 301,788 and for new onset depression n = 30,877 with diabetes excluded); the association between history of depression and the 12 CVD endpoints as well as between new onset depression and the 12 CVD endpoints remained strong, and the magnitude of HRs was not significantly altered after adjustment for age, gender, and GP practice. (Results not shown) In addition, adjustment for body mass index (as well as cardiovascular risk factors) produced estimates of equal magnitude for the association of history of depression and the 12 outcomes, as well as for new onset depression and the 12 outcomes. (Table B in S1 File)

## Discussion

This linked electronic health record study of 1,937,360 patients shows that GP-diagnosed depression is associated with an increased risk of a wide range of pathologically heterogeneous cardiac, cerebrovascular, and peripheral vascular diseases in people who were free of cardiovascular disease at baseline. These associations were of similar magnitude across these 12 diseases, which included those previously studied (such as heart attack and ischemic stroke), as well as diseases for which there has been little or no previous literature such as stable angina, heart failure, sudden cardiac death, transient ischemic attack, abdominal aortic aneurysm, and peripheral arterial disease. These findings support the theories of complex multifactorial mechanisms in the relationship between depression and cardiovascular disease. Additionally, our results highlight the importance of assessing and managing global cardiovascular risk to prevent cardiovascular diseases in people with depression.

Clinicians manage a large group of patients who have a history of depression (19% of our sample) and a smaller group with new onset depression (2.9%). To our knowledge, this is the first large-scale study to examine GP-diagnosed new onset depression as a risk factor of cardiovascular disease, as the majority of previous studies[4–6,10] assessed prevalent depression. We hypothesized that new onset depression may show stronger associations for certain cardiovascular endpoints compared to history of depression (analogous to stronger effects for current as compared to ex-smokers).[24] While a history of depression points to cumulative, perhaps atherogenic effects, new onset depression may be related to acute events, such as diminished heart rate variability. We did not observe differential associations with new onset depression.

In this study we contribute more cardiovascular events (greater than 90,000) than any previous meta-analysis. For example, a meta-analysis of coronary heart disease found 4,016 events in 21 studies[7], and a meta-analysis of stroke found 8,478 events in 28 studies[5]. Previous studies have attempted to investigate sex differences[4,25] in the depression-CVD association. Ours is the first to have the statistical power to reliably demonstrate that the associations do not differ in women and men. This is clinically relevant as new onset depression was more common in women (63.9% of cases) than in men.

We separated out a wider range of cardiac diseases than previous studies and found that GP-diagnosed depression was strongly associated with incident stable angina and incident unstable angina, common initial manifestations of cardiovascular disease. Previous studies have either included angina as a component of aggregate coronary heart disease outcomes along with myocardial infarction and ischemic heart disease,[6,8] or have excluded [8] stable angina on the basis that people with mood disorders are more likely to report angina-like chest pain in the absence of narrowed coronary arteries. However, research suggests that this phenomenon may only occur in depression with co-morbid panic disorders.[26] In our study the association between new onset depression and initial presentation of myocardial infarction (MI) became insignificant after adjustment for cardiovascular risk factors, but remained significant for history of depression. This is in line with a recent meta-analysis[6], which found a 60% increased risk of myocardial infarction in depressed participants; the pooled estimate was based on 8 studies, of which only 3 excluded prevalent CVD, while the rest showed non-significant associations, were based on elderly populations, or on self-reported MI with small sample sizes. In other meta-analyses,[7,8] fatal and non-fatal MI were grouped with coronary heart disease death into a single outcome, making comparison with our results difficult.

We found that depression was associated with elevated risk of heart failure, an increasingly common initial presentation of cardiovascular disease. Few other cohort studies have examined this association. A cohort of elderly (>70 years) participants found that depression was associated with increased risk of developing heart failure among women only.[27] We add to current literature by showing that depression may contribute to the development of heart failure not only in susceptible, but also in disease-free populations.

We found that depression was associated with incident major, but uncommon, cerebrovascular events (sub-arachnoid haemorrhage, intracerebral haemorrhage) and more common events (such as transient ischaemic attack). These are novel findings; none of these subtypes of cerebrovascular disease was reported in a recent meta-analysis of 28 cohort studies of depression and cerebrovascular disease;[5] based on pooled estimates across 6 cohorts the meta-analysis found [5] an association for ischemic stroke of 1.25 (95%CI 1.11–1.40), which overlaps with our estimates (new onset depression: HR = 1.18, 0.98–1.42; history of depression: 1.31, 1.23–1.40). We found significantly elevated risk of haemorrhagic stroke among those with history of depression, which may be explained by the larger (9-fold) number of people with a history of depression compared to new onset depression. The risk of incident transient ischemic attack was 35% higher among depressed individuals, which has not been previously demonstrated. Certain meta-analyses [6] and prospective cohort studies[11] aggregated stroke subtypes due to insufficient numbers, while other studies loosely defined the composition of their total cerebrovascular endpoints,[12] or only considered one type of stroke,[10] potentially preventing observation of differential associations with depression.

To our knowledge there have not been large scale prospective studies examining the association of GP-diagnosed depression with subsequent peripheral arterial disease or abdominal aortic aneurysm in initially healthy populations. Our study shows that depression confers an elevated risk of initial presentation with peripheral arterial disease, after adjustment for cardiovascular risk factors including smoking. History of depression was also associated with abdominal aortic aneurysm. Until recently, abdominal aortic aneurysm was considered a manifestation of atherosclerosis but the latest evidence suggests an independent, non-atherosclerotic pathogenesis and a stronger effect of smoking on vascular disease in the peripheral arteries.[28] Our findings demonstrate a novel association of depression with a vascular condition, which is pathologically distinct from thrombotic coronary heart disease.

### Underlying bio-behavioural pathways

Cardiovascular risk factors demonstrate somewhat specific associations with different cardiovascular diseases; for example, people with type II diabetes have been shown to be at lower risk of subarachnoid haemorrhage, transient ischaemic attack,[29] and risk of abdominal aortic aneurysm[30], compared to people without diabetes; blood pressure shows markedly different associations with peripheral arterial disease (strong associations with systolic, weaker with diastolic blood pressure) and abdominal aortic aneurysm (strong associations with diastolic, weak or non-existent associations with systolic blood pressure[31]). Our findings underscore the stability of the relationship between depression and cardiovascular disease, and the need for further exploration of underlying mechanisms. The relationship between CVD and depression is likely to be complex, and the bi-directional association between the conditions is important. For example, lower ‘cardiovascular fitness’ predicts incident depression, which can subsequently influence the incidence of CVD per se.(30) All these outcomes, behaviours, and physiological responses are likely to be interrelated and have common mediators. The pathways may involve differing simultaneous mechanisms, so that simplistic explanations of the relationship may be unhelpful.(30) As well as behavioural and physiological explanations, there is evidence that health behaviours may also differ in people with depression. Smokers with depression may be less likely to engage in smoking cessation, despite the fact that smoking cessation is associated with an improvement in mood for those with and without psychiatric diagnoses.(31) There is also evidence that people with depression and CVD risk factors such as diabetes are less likely to adhere to screening regimes or adhere to interventions to reduce excess CVD risk such as lipid lowering medications.(32) Individuals with a depressive episode are also more likely to be unemployed, belong to the most deprived quintiles, have low educational qualifications, and unstable housing.[32] In our study, however, adjustment for socioeconomic status and cardiovascular risk factors, including smoking, did not significantly alter our estimate sizes.

### Limitations

Each linked electronic health record (EHR) source used has its own limitations, discussed in detail elsewhere.[16] Missing data for potential confounding or mediating factors such as alcohol use and ethnicity necessitated a complete case analysis, which suggested that these factors were unlikely to explain much of the observed associations. The data are limited to GP-recorded depression, which reflect incidence, presentation, and recording by GPs. There is a lack of validation work on depression recording in primary care databases, and evidence suggests that a GP diagnosis of depression has a specificity of 81.3% and a sensitivity of around 50%.[33] Some individuals labelled in their records with depression may not meet criteria for a major depressive episode. This misclassification bias may lead to an underestimation the observed association. Individuals with depression in this study had a higher rate of primary care consultations in the year before study entry compared to those without. We cannot exclude that the frequency of consultation meant that depressed patients were more likely to be diagnoses with cardiovascular conditions nor can we exclude that they were seen more frequently because they were developing symptoms of cardiovascular disease. A recent paper using CALIBER data identified that patients who went on to develop myocardial infarction had increasing consultations for chest pain in the months prior to their MI.[34] We cannot address the extent to which the association between depression and cardiovascular disease could be mediated through factors not recorded in EHR sources, such as physical activity or diet. Similarly to national primary care record sources in other countries, we are not able to obtain imaging results which would allow better phenotypic resolution of different disease endpoints (e.g. distinguish heart failure with and without preserved ejection fraction). Despite these important limitations the EHR data has an important strength—these are the data used in clinical practice to make decisions.

### Implications

Almost 19% of patients in our population-based study were diagnosed by their GP as having a history of depression and were at increased risk of all cardiovascular diseases. As the prevalence and incidence of depression and cardiovascular disease remain high, our observed associations have clinical importance. Trial evidence is lacking that treatment of depression reduces risk of cardiac or other cardiovascular endpoints.[35] However there are recent recommendations from the American Heart Association and American College of Cardiology[36] and from the Joint British Societies[37] that cardiovascular risk should be estimated in apparently healthy individuals in order to inform the initiation of preventive medications, such as statins. While some guidelines for the management of depression recommend assessment of cardiovascular risk (confined to patients prescribed specific antidepressant drugs[38]), other guidelines[35,39] on cardiovascular disease prevention are not explicit about management of cardiovascular risk among individuals with depression. Our findings provide some support for systematic assessment of the overall cardiovascular risk in patients with depression. Our results also have implications for the design and interpretation of randomised trials on depression and cardiovascular disease. Since depression was non-specifically associated with many cardiovascular diseases, clinical trials may benefit from considering inclusion of primary endpoints combining all cardiovascular diseases, rather than focusing on fatal coronary heart disease and non-fatal myocardial infarction (as in the ENRICHD trial).[35]

In conclusion, this large linked electronic health record study provides strong evidence that depression is a risk factor for the initial presentation of a range of cardiac, cerebrovascular, and peripheral arterial diseases, with no evidence of differential effects by gender.

## Supporting Information

S1 File **Text A. Definition of depression using the CPRD. Table A. Overview of codes and data sources used to define each cardiovascular endpoints. Text B. Multiple imputation.** Fig A. Hazard ratios (HR) and 95% confidence interval (95%CI) for the association of new onset depression with 12 cardiovascular diseases, adjusted for age, sex, smoking, systolic blood pressure, diabetes, cholesterol, and socio-economic status (63,761 events in 629,659 men and 604,278 women). **Fig B. Hazard ratios (HR) and 95% confidence interval (95%CI) for the association of** **history depression** **with 12 cardiovascular diseases, restricted to patients with recorded data (complete case) for CVD risk factors, ethnicity, and alcohol abuse (n = 1,018,538). Fig C. Hazard ratios (HR) and 95% confidence interval (95%CI) for the association of** **new onset depression** **at baseline with 12 cardiovascular diseases, restricted to patients with recorded data (complete case) for CVD risk factors, ethnicity, and alcohol abuse only (n = 674,931). Table B. Hazard ratios (HR) and 95% confidence interval (95%CI) for the association of: (a.) history of depression and (b.) new onset depression at baseline with 12 cardiovascular diseases, adjusted for all risk factors (age, sex, smoking, systolic blood pressure, diabetes, cholesterol, socioeconomic factors)** **plus body mass index (BMI).**
(DOCX)
