# Supplementary material for: Depression as a Risk Factor for the Initial Presentation of Twelve Cardiac, Cerebrovascular, and Peripheral Arterial Diseases: Data Linkage Study of 1.9 Million Women and Men
Source: PLoS One. 2016 Apr 22;11(4):e0153838. doi: 10.1371/journal.pone.0153838 (PMC4841529; doi:10.1371/journal.pone.0153838)
Supplement: S1 File — Text A. Definition of depression using the CPRD. Table A. Overview of codes and data sources used to define each cardiovascular endpoints. Text B. Multiple imputation. Fig A. Hazard ratios (HR) and 95% confidence interval (95%CI) for the association of new onset depression with 12 cardiovascular diseases, adjusted for age, sex, smoking, systolic blood pressure, diabetes, cholesterol, and socio-economic status (63,761 events in 629,659 men and 604,278 women). Fig B. Hazard ratios (HR) and 95% confidence interval (95%CI) for the association of history depression with 12 cardiovascular diseases, restricted to patients with recorded data (complete case) for CVD risk factors, ethnicity, and alcohol abuse (n = 1,018,538). Fig C. Hazard ratios (HR) and 95% confidence interval (95%CI) for the association of new onset depression at baseline with 12 cardiovascular diseases, restricted to patients with recorded data (complete case) for CVD risk factors, ethnicity, and alcohol abuse only (n = 674,931). Table B. Hazard ratios (HR) and 95% confidence interval (95%CI) for the association of: (a.) history of depression and (b.) new onset depression at baseline with 12 cardiovascular diseases, adjusted for all risk factors (age, sex, smoking, systolic blood pressure, diabetes, cholesterol, socioeconomic factors) plus body mass index (BMI). (DOCX) [file pone.0153838.s001.docx]

**Supplemental Information (S1 File)**

**Daskalopoulou M, George J, Walters K, et al. Depression as a risk factor for the initial presentation of twelve cardiac, cerebrovascular, and peripheral arterial diseases: data linkage study of 1.9 million women and men.**

**Text A in S1 File.** **Definition of depression using the CPRD**

**Table A in S1 File. Overview of codes and data sources used to define each cardiovascular endpoints.**

**Text B in S1 File. Multiple imputation**

Figure A in S1 File. Hazard ratios (HR) and 95% confidence interval (95%CI) for the association of new onset depression with 12 cardiovascular diseases, adjusted for age, sex, smoking, systolic blood pressure, diabetes, cholesterol, and socio-economic status (63,761 events in 629,659 men and 604,278 women)

**Figure B in S1 File. Hazard ratios (HR) and 95% confidence interval (95%CI) for the association of history depression with 12 cardiovascular diseases, restricted to patients with recorded data (complete case) for CVD risk factors, ethnicity, and alcohol abuse (n=1,018,538)**

**Figure C in S1 File. Hazard ratios (HR) and 95% confidence interval (95%CI) for the association of new onset depression at baseline with 12 cardiovascular diseases, restricted to patients with recorded data (complete case) for CVD risk factors, ethnicity, and alcohol abuse only (n=674,931)**

**Table B in S1 File. Hazard ratios (HR) and 95% confidence interval (95%CI) for the association of: (a.) history of depression and (b.) new onset depression at baseline with 12 cardiovascular diseases, adjusted for all risk factors (age, sex, smoking, systolic blood pressure, diabetes, cholesterol, socioeconomic factors) plus body mass index (BMI)**

**Text A in S1 File.** **Definition of depression using the CPRD**

Diagnosis of depression recorded in the CPRD was defined using the Read codes listed in the supplement for the paper by Rait et al (Rait G, Walters K, Griffin M, Buszewicz M, Petersen I, Nazareth I. Recent trends in the incidence of recorded depression in primary care. *Br.J.Psychiatry* 2009;**195**:520-4.) and supplemented by our own list of Read codes (shown below), which included those from the administration domain (starting with 9) and other therapeutic procedures domain (starting with 8).

Depression was coded into the following categories:

0 – not diagnosed

1 – history of depression

2 – possible depression

3 – dysthymia

4 – diagnosed with depression single episode (SE) or recurrence not specified (NS)

5 – recurrent depression code

6 – secondary depression

7 – depression with anxiety

8 – depression with agitation

9 – other or unspecified

The variable was then dichotomised into evidence of depression or no evidence of depression, with the addition of antidepressants (see below).


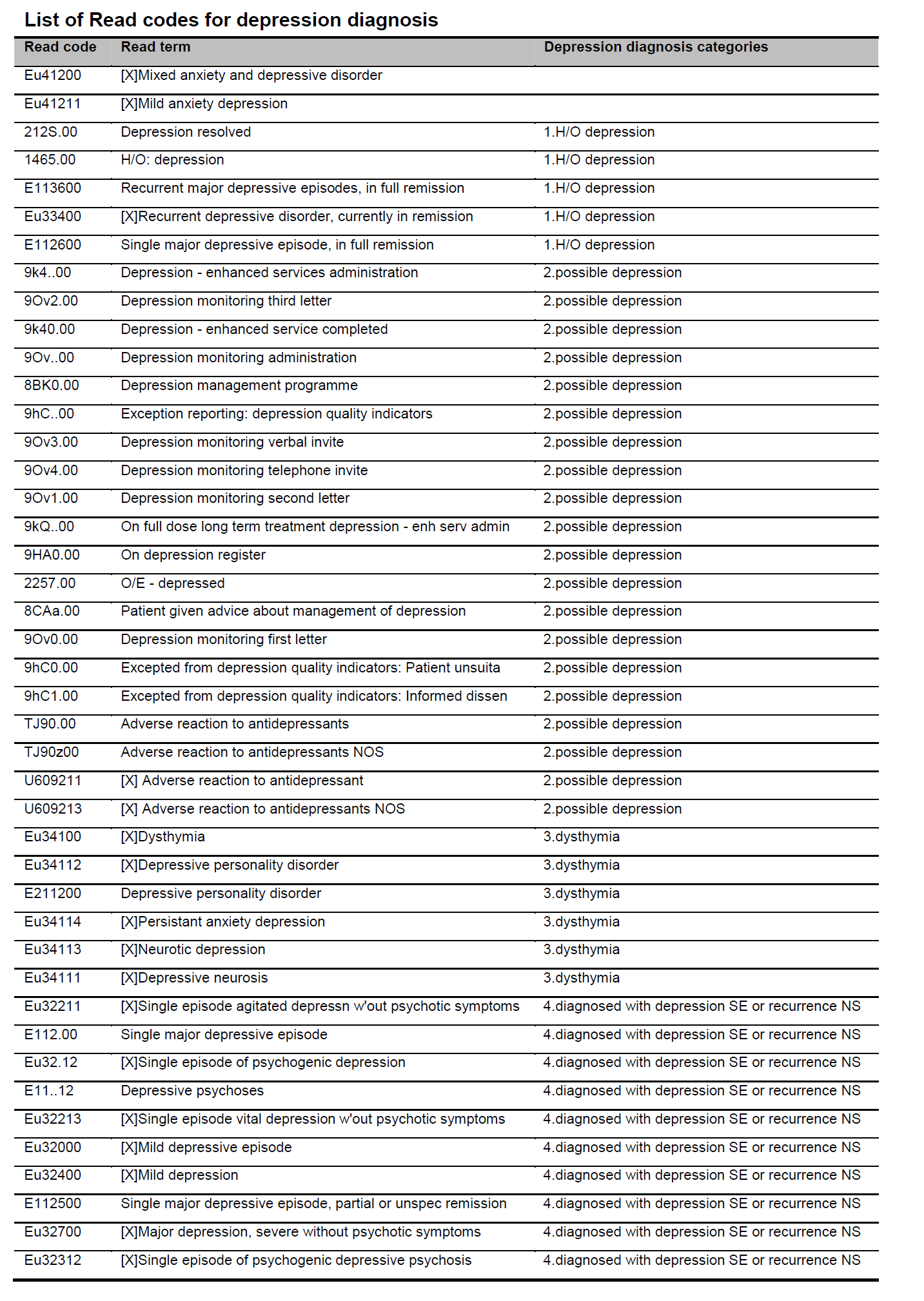

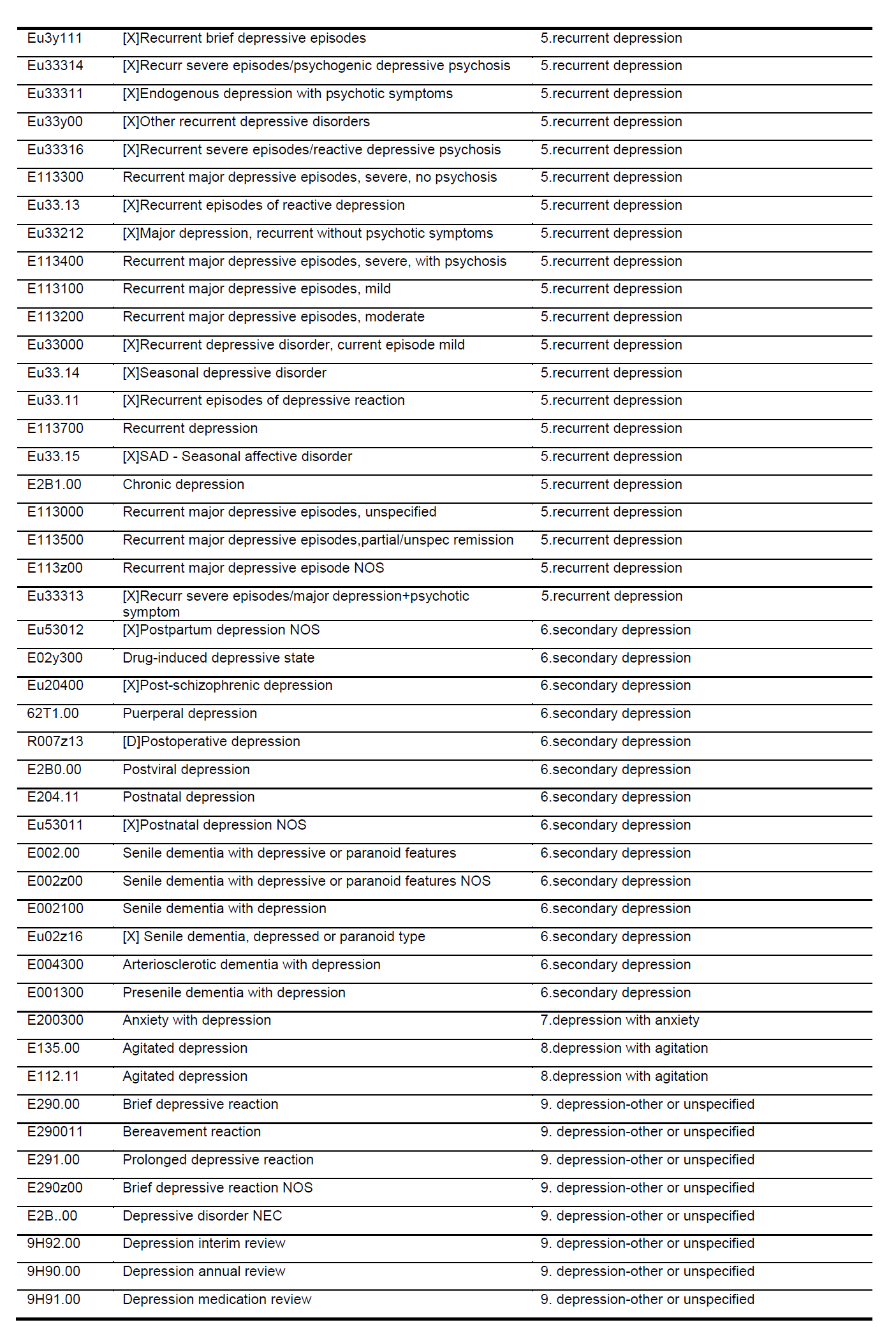


The depression diagnosis variable was supplemented by the addition of any prescription for any of the below reported antidepressants, as based on the British National Formulary.


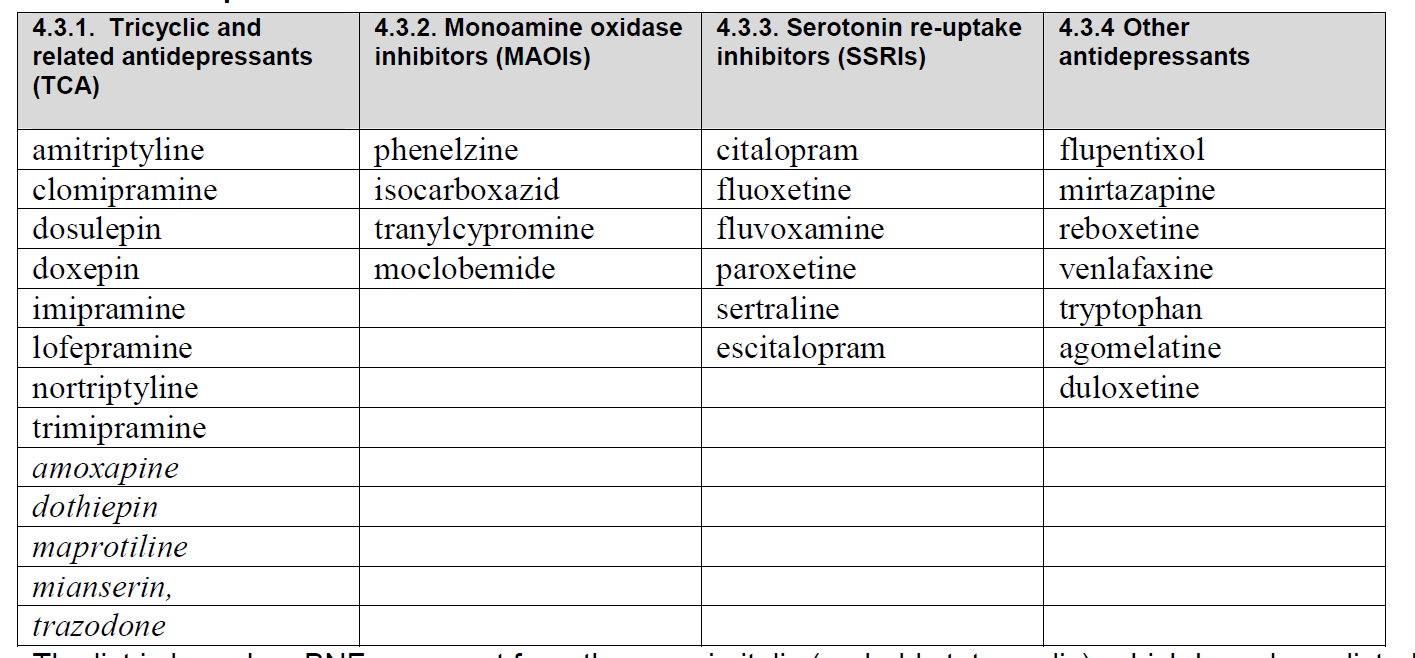


**Table A in S1 File. Overview of codes and data sources used to define each cardiovascular endpoints.**

| **Endpoint** | **CPRD – Read codes** | **MINAP – specific disease registry** | **HES – OPCS 4 hospital procedures** | **HES – ICD 10 hospital diagnoses**† | **ONS – ICD 10 causes of death**‡ |
| --- | --- | --- | --- | --- | --- |
| **Stable angina** | G33..00: Stable Angina.  G33z.00: Angina pectoris NOS + 25 other codes for diagnosis of stable angina pectoris.  30 codes for evidence of coronary artery disease at angiography (CT,MR, invasive or not specified).  151 Read codes for evidence of myocardial ischaemia (Resting ECG, exercise ECG, stress echo, radioisotope scan).  Two or more successive prescriptions for anti-anginals. | nu | K40-K46: Coronary artery bypass graft.  K49,K50 and K75: Percutaneous coronary intervention, not within 30 days of an acute coronary syndrome. | I20: Stable angina pectoris excluding unstable angina (I20.0). | nu |
| **Unstable angina** | G311.13/G311100: Unstable angina.  G233200: Angina at rest.  G311400: Worsening angina + 13 other codes. | Discharge diagnosis of unstable angina, no raised ST elevation.  No raised troponin levels. | nu | I20.0: Unstable or worsening angina.  I24: Acute ischaemic heart disease.  I24.0: Coronary thrombosis not resulting in MI.  I24.8: Other forms of ischaemic heart disease.  I24.9: Acute ischaemic heart disease, unspecified. | nu |
| **Coronary heart disease not otherwise specified** | G3…00: Ischaemic heart disease + 90 other codes including CHD NOS, chronic ischaemic heart disease, silent myocardial infarction. | nu | nu | CHD NOS, chronic ischaemic heart disease, silent MI (I25) excluding.I25.2, old MI. | nu |
| **Acute Myocardial Infarction (MI)** | G30X000: Acute ST segment elevation myocardial infarction.  G307100: Acute non-ST segment elevation myocardial infarction.  G30..14: Heart attack.  G30..15: MI Acute myocardial infarction + 60 other codes as Acute MI not otherwise specified. | MI with or without ST elevation based on initial electrocardiogram findings, raised troponins and clinical diagnosis. | nu | I21: Acute myocardial infarction.  I23: Current complications of acute MI. | nu |
| **Unheralded coronary death** | Any CVD excluded. | Any CVD excluded. | Any CVD excluded. | Any CVD excluded. | I20: Angina Pectoris.  I21: Acute MI.  I22: Subsequent MI.  I23: Certain current complications following acute MI.  I24: Other acute ischaemic heart diseases.  I25: Chronic ischaemic heart disease. |
| **Heart failure** | G58..00: Heart Failure + 92 other Read codes for heart failure diagnosis. | nu | nu | I50: Heart failure.  I11.0: Hypertensive heart disease with (congestive) heart failure.  I13.0: Hypertensive heart and renal disease with (congestive) heart failure.  I13.2: Hypertensive heart and renal disease with both (congestive) heart failure and renal disease. | I50 Heart failure.  I11.0 Hypertensive heart disease with (congestive) heart failure.  I13.0: Hypertensive heart and renal disease with (congestive) heart failure.  I13.2: Hypertensive heart and renal disease with both (congestive) heart failure and renal disease |
| **Ventricular arrhythmias, cardiac arrest and sudden cardiac death** | G574.00: Ventricular fibrillation and flutter.  G757.00: Cardiac arrest + 35 other Read codes for ventricular fibrillation, asystole, cardiac arrest, cardiac resuscitation, electro-mechanical dissociation.  G575100: Sudden cardiac death. | nu | X50: Implanted cardiac defibrillation device.  K59: Implantation, revision and renewal of cardiac defibrillator. | I46: Cardiac arrest.  I47.0: Re-entry ventricular arrhythmia.  I47.2: Ventricular tachycardia. | I46: Cardiac arrest.  I47.0: Re-entry ventricular arrhythmia.  I47.2: Ventricular tachycardia. |
| **Transient ischaemic attack** | Fyu5500: [X]Other transient cerebral ischaemic attacks + related symptoms + 5 other Read codes. | nu | nu | G458: Other transient cerebral ischaemic attacks and related syndromes.  G459: Transient cerebral ischaemic attack, unspecified. | nu |
| **Ischaemic stroke** | G64..11: CVA – cerebral artery occlusion, G64..13 Stroke due to cerebral arterial occlusion.  G6W..00: Cerebral infarction due to unspecified occlusion/stenosis of precerebral arteries.  G6X..00: Cerebral infarction due to unspecified occlusion/stenosis of cerebral arteries plus 8 other codes. | nu | Stroke NOS with carotid endarterectomy or stenting within 90 days (OPCS codes L294, L295, L311, L314; Read codes 7A20300 + 4 others). | I63: Cerebral infarction. | I63: Cerebral infarction. |
| **Subarachnoid haemorrhage** | G601.00:Subarachnoid haemorrhage from carotid siphon and bifurcation.  G602.00: Subarachnoid haemorrhage from middle cerebral artery.  G60X.00: Subarachnoid haemorrhage from intracranial artery, unspecified. | nu | nu | I60: Subarachnoid haemorrhage. | I60: Subarachnoid haemorrhage. |
| **Intracerebralhaemorrhage** | Gyu6F00: [x] Intracerebral haemorrhage in hemisphere, unspecified + 16 other codes. | nu | nu | I61: Intracerebral haemorrhage. | I61: Intracerebral haemorrhage. |
| **Stroke not otherwise specified** | G66..11: Cerebrovascular accident unspecified + 14 other Read codes. | nu | U54.3: Delivery of rehabilitation for stroke. | I64: Stroke not specified as haemorrhage or infarction.  G463-G467: Stroke syndromes. | I64: Stroke not specified as haemorrhage or infarction.  I672: Cerebral atherosclerosis.  I679: Cerebrovascular disease, unspecified. |
| **Peripheral arterial disease** | 63 codes for lower limb peripheral arterial disease diagnosis (including diabetic PAD, gangrene, arterial thrombosis of the leg and intermittent claudication).  Evidence of atherosclerosis of iliac and lower limb arteries based on angiography or Dopplers. | nu | L50-L54: Bypass, reconstruction and other open operations on iliac artery.  L58-L60, L62: Bypass, reconstruction, transluminal operations or other open operations of femoral artery.  L65: Revision of reconstruction of artery. | I70.2: atherosclerosis of arteries of extremities.  I73.9: Peripheral vascular disease intermittent claudication  E10.05,E11-E14: Peripheral complications of diabetes including gangrene, insulin dependent diabetes mellitus, non-insulin-dependent diabetes mellitus, malnutrition-related diabetes mellitus, other specified diabetes mellitus, unspecified diabetes mellitus. | I70.2: Atherosclerosis of arteries of extremities.  I73.9: Peripheral vascular disease intermittent claudication.  Peripheral complications of diabetes including gangrene 0.5 suffix of E10: Insulin dependent diabetes mellitus, E11: Non-insulin-dependent diabetes mellitus, E12: Malnutrition-related diabetes mellitus, E13: Other specified diabetes mellitus, E14: Unspecified diabetes mellitus |
| **Abdominal aortic aneurysm** | G714.00: AAA without mention of rupture + 12 more codes for AAA diagnosis.  42 codes for AAA procedures. | nu | L16: Extra anatomic bypass of aorta.  L18-L23: Replacement of aneurysmal segment of aorta, bypass of segment of aorta, plastic repair of aorta.  L25-L28: Transluminal or endovascular insertion of stent on aneurysmal segment of aorta. | I71.3: Ruptured AAA.  171.4: AAA without rupture.  I71.5: Ruptured thoraco-abdominal aortic aneurysm.  I71.6: Thoracoabdominal aortic aneurysm without mention of rupture.  I71.8: Aortic aneurysm of unspecified site, ruptured.  I71.9: Aortic aneurysm of unspecified site, without mention of rupture. | I71.3: Ruptured AAA.  I71.4: AAA without rupture.  I71.5: Ruptured thoraco-abdominal aortic aneurysm.  I71.6: Thoracoabdominal aortic aneurysm without mention of rupture.  I71.8: Aortic aneurysm of unspecified site, ruptured.  I71.9: Aortic aneurysm of unspecified site, without mention of rupture. |

Note: AAA, aortic abdominal aneurysm; CVD, cardiovascular disease; MI, myocardial infarction; NOS, not otherwise specified; nu = not used in definition; OPCS, Office of Population Censuses and Surveys Classification of Interventions and Procedures. †Primary cause of admission. ‡Underlying cause of death.

**Text B in S1 File. Multiple imputation**

Multiple imputation was implemented using the *mice* algorithm in the statistical package R. Imputation models were estimated separately for men and women and included:

a) All baseline covariates used in the main analysis (age, quadratic age, diabetes, smoking, systolic blood pressure, total cholesterol, HDL cholesterol, index of multiple deprivation),

b) Prior (between 1 and 4 years before study entry) and post (between 0 and 1 year after study entry) averages of continuous covariates in the main analysis,

c) Baseline measurements of covariates not part of the main analysis (diastolic blood pressure, alcohol intake, white cell count, haemoglobin, creatinine, alanine transferase),

d) Baseline medications (statins, blood pressure medications, low-dose aspirin, loop diuretics, oral contraceptives and hormone replacement therapy),

e) Coexisting medical conditions (history of depression, cancer, renal disease, liver disease and chronic obstructive pulmonary disease),

f) The Nelson-Aalen hazard and the event status for each endpoint analysed in the data^2^.

Non-normally distributed variables were log-transformed for imputation and exponentiated back to their original scale for analysis. Five multiply imputed datasets were generated, and Cox models fitted to each dataset. Coefficients were combined using Rubin’s rules. We used the Kolmogorov-Smirnov test to compare the distribution of observed versus imputed log-transformed covariates.

Figure A in S1 File. Hazard ratios (HR) and 95% confidence interval (95%CI) for the association of new onset depression with 12 cardiovascular diseases, adjusted for age, sex, smoking, systolic blood pressure, diabetes, cholesterol, and socio-economic status (63,761 events in 629,659 men and 604,278 women)


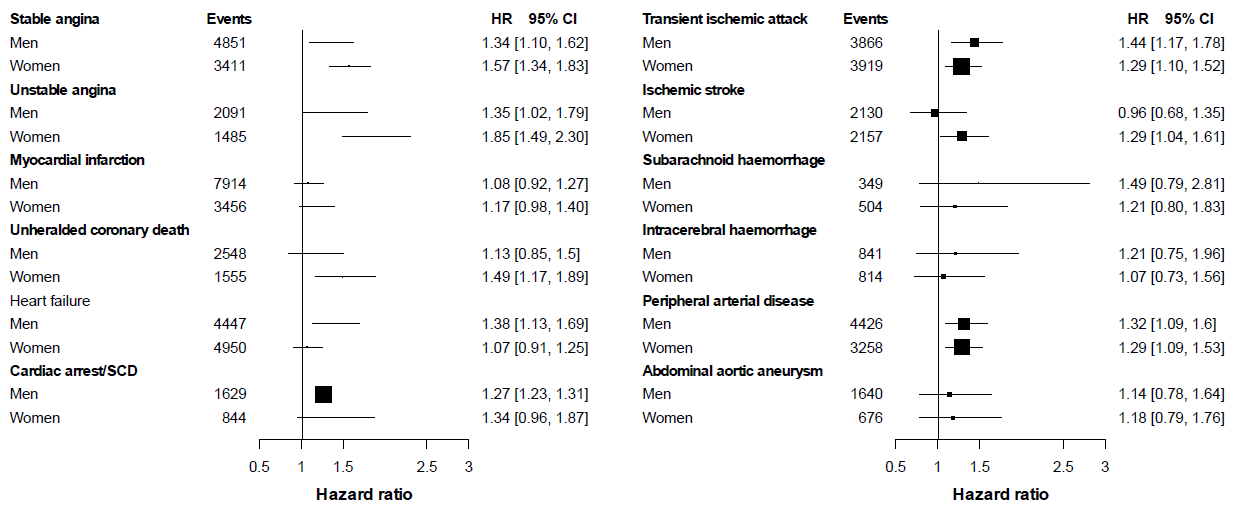


Abbreviation: SCD; sudden cardiac death

**Figure B in S1 File. Hazard ratios (HR) and 95% confidence interval (95%CI) for the association of history depression with 12 cardiovascular diseases, restricted to patients with recorded data (complete case) for CVD risk factors, ethnicity, and alcohol abuse (n=1,018,538)***
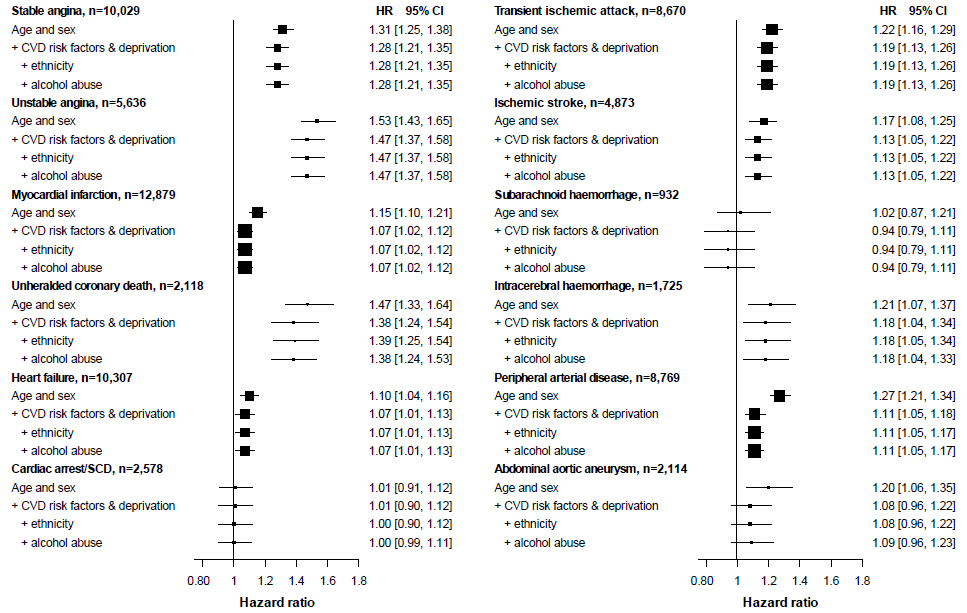


*First row: Adjusted for age and sex. Second row: Adjusted for age, sex, cardiovascular risk factors (smoking, systolic blood pressure, cholesterol, diabetes) and socioeconomic status. Third row: Adjusted for age, sex, CVD risk factors, socioeconomic status, and ethnicity. Fourth row: Adjusted for age, sex, CVD risk factors, socioeconomic status, and heavy alcohol consumption

**Figure C in S1 File. Hazard ratios (HR) and 95% confidence interval (95%CI) for the association of new onset depression at baseline with 12 cardiovascular diseases, restricted to patients with recorded data (complete case) for CVD risk factors, ethnicity, and alcohol abuse only (n=674,931)***


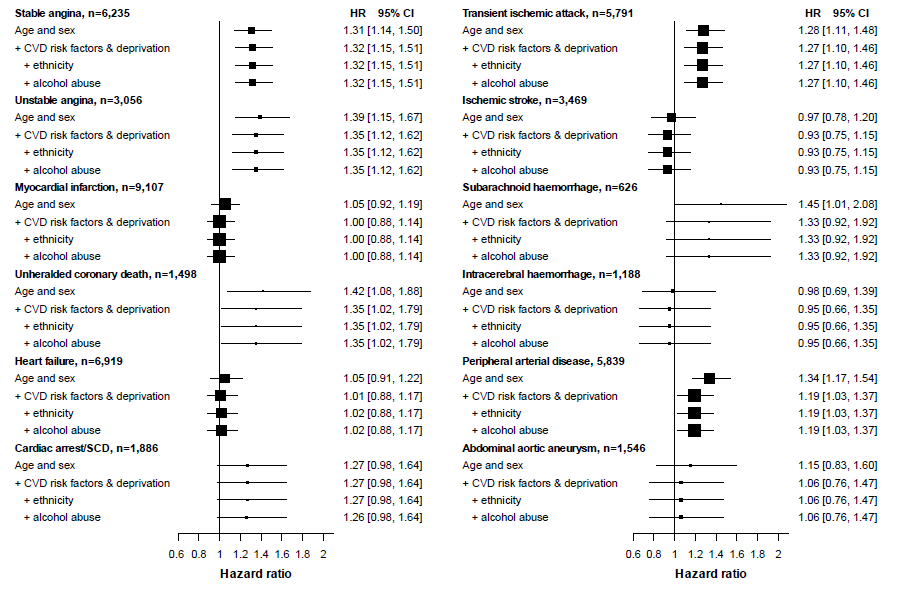
 * First row: Adjusted for age and sex. Second row: Adjusted for age, sex, cardiovascular risk factors (smoking, systolic blood pressure, cholesterol, diabetes) and socioeconomic status. Third row: Adjusted for age, sex, CVD risk factors, socioeconomic status, and ethnicity. Fourth row: Adjusted for age, sex, CVD risk factors, socioeconomic status, and heavy alcohol consumption

**Table B in S1 File. Hazard ratios (HR) and 95% confidence interval (95%CI) for the association of: (a.) history of depression and (b.) new onset depression at baseline with 12 cardiovascular diseases, adjusted for all risk factors (age, sex, smoking, systolic blood pressure, diabetes, cholesterol, socioeconomic factors) plus body mass index (BMI)**

|  | **(a.) History of depression (367,117/1,937,360)** | | | **(b.) New onset depression (39,747/1,356,578)** | | |
| --- | --- | --- | --- | --- | --- | --- |
| **Endpoint** | **n events** | **HR*** | **95%CI** | **n events** | **HR*** | **95%CI** |
| **Coronary** |  |  |  |  |  |  |
| Stable angina | 13,221 | 1.38 | 1.32-1.44 | 8,262 | 1.46 | 1.30-1.65 |
| Unstable angina | 5,636 | 1.70 | 1.59-1.81 | 3,576 | 1.61 | 1.35-1.91 |
| Myocardial infarction | 16,239 | 1.21 | 1.16-1.27 | 11,370 | 1.11 | 0.99-1.30 |
| Unheralded CHD death | 5,515 | 1.23 | 1.14-1.32 | 4,103 | 1.30 | 1.08-1.56 |
| **Other myocardial** |  |  |  |  |  |  |
| Heart failure | 14,359 | 1.17 | 1.12-1.23 | 9,397 | 1.15 | 1.01-1.30 |
| Cardiac arrest/sudden cardiac death | 3,375 | 1.13 | 1.03-1.24 | 2,473 | 1.31 | 1.03-1.66 |
| **Cerebrovascular** |  |  |  |  |  |  |
| Transient ischaemic stroke (TIA) | 11,714 | 1.31 | 1.25-1.38 | 7,785 | 1.35 | 1.19-1.53 |
| Ischaemic stroke | 6,053 | 1.26 | 1.17-1.34 | 4,287 | 1.17 | 0.97-1.41 |
| Subarachnoid haemorrhagic stroke | 1,278 | 1.18 | 1.02-1.36 | 853 | 1.30 | 0.92-1.85 |
| Intracerebral haemorrhage | 2,388 | 1.31 | 1.18-1.46 | 1,655 | 1.13 | 0.84-1.52 |
| **Abdominal and lower limb** |  |  |  |  |  |  |
| Peripheral arterial disease | 11,519 | 1.24 | 1.18-1.30 | 7,684 | 1.31 | 1.15-1.49 |
| Abdominal aortic aneurysm | 3,135 | 1.12 | 1.01-1.24 | 2,316 | 1.15 | 0.88-1.52 |
| *Models adjusted for age (linear and quadratic), systolic blood pressure, total cholesterol, high density lipoprotein, diabetes, body mass index, index of multiple deprivation score, smoking, and stratified by GP practice and gender. | | | | | | |
